# *In vivo* immuno-targeting of an extracellular epitope of membrane bound preferentially expressed antigen in melanoma (PRAME)

**DOI:** 10.18632/oncotarget.19579

**Published:** 2017-07-26

**Authors:** Dmitry Pankov, Ludvig Sjöström, Teja Kalidindi, Sang-Gyu Lee, Kjell Sjöström, Rui Gardner, Michael R. McDevitt, Richard O’Reilly, Daniel L.J. Thorek, Steven M. Larson, Darren Veach, David Ulmert

**Affiliations:** ^1^ Immunology Program, Department of Pediatrics, Memorial Sloan Kettering Cancer Center, New York, NY, USA; ^2^ Flow Cytometry Core Facility, Memorial Sloan Kettering Cancer Center, New York, NY, USA; ^3^ Molecular Pharmacology Program, Memorial Sloan Kettering Cancer Center, New York, NY, USA; ^4^ Division of Oncology, Clinical Sciences, Lund University and Skåne University Hospital, Lund, Sweden; ^5^ Department of Radiology, Memorial Sloan Kettering Cancer Center, New York, NY, USA; ^6^ Department of Radiology, Weill Cornell Medical College, New York, NY, USA; ^7^ Division of Nuclear Medicine and Molecular Imaging, Department of Radiology and Radiological Science, Sidney Kimmel Comprehensive Cancer Center, Johns Hopkins School of Medicine, Baltimore, MD, USA; ^8^ Bone Marrow Transplant Service, Memorial Sloan Kettering Cancer Center, New York, NY, USA; ^9^ Innovagen AB, Lund, Sweden; ^10^ Department of Medicine, Weill Cornell Medical College, New York, NY, USA; ^11^ Cancer Molecular and Functional Imaging Program, Department of Oncology, Sidney Kimmel Comprehensive Cancer Center, Johns Hopkins School of Medicine, Baltimore, MD, USA

**Keywords:** PRAME, immuno-targeting, cancer, noninvasive imaging, targeted therapy

## Abstract

Preferentially Expressed Antigen in Melanoma (PRAME) is a cancer/testis antigen that is overexpressed in a broad range of malignancies, while absent in most healthy human tissues, making it an attractive diagnostic cancer biomarker and therapeutic target. Although commonly viewed as an intracellular protein, we have demonstrated that PRAME has a membrane bound form with an external epitope targetable with conventional antibodies. We generated a polyclonal antibody (Membrane associated PRAME Antibody 1, MPA1) against an extracellular peptide sequence of PRAME. Binding of MPA1 to recombinant PRAME was evaluated by Enzyme-Linked Immunosorbent Assay (ELISA). Flow cytometry and confocal immunofluorescence microscopy of MPA1 was performed on multiple tumor cell lines. Reverse Transcription Polymerase Chain Reaction (RT-PCR) for *PRAME* was conducted to compare protein and transcriptional expression levels. We demonstrated a robust proof-of-concept for PRAME targeting *in vivo* by radiolabeling MPA1 with zirconium-89 (^89^Zr-DFO-MPA1) and demonstrating high specific uptake in PRAME expressing tumors. To our knowledge, this is the first time a cancer testis antigen has been targeted using conventional antibody technologies. Thus, PRAME can be exploited for multiple clinical applications, including targeted therapy, diagnostic imaging and treatment guidance in a wide-range of malignancies, with minimal off-target toxicity.

## INTRODUCTION

**Figure 1:**
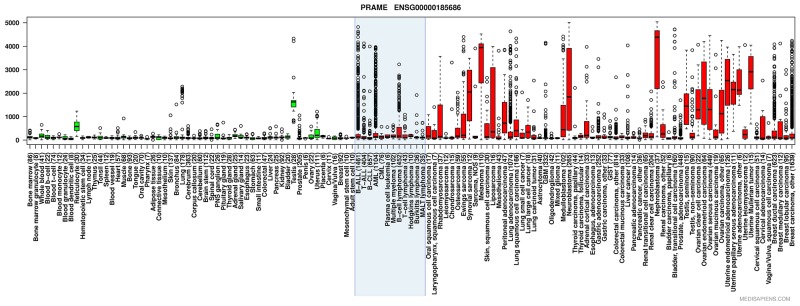
Anatomical and disease specific *PRAME* expression in various types of normal and malignant tissues Box plot with whiskers describing relative *PRAME* expression in healthy (green boxes) and malignant (red boxes) tissues. Boxes include values between the upper and lower quartile, with whiskers extending to 1.5 times the interquartile ranges. Outliers are marked as hollow circles. The blue shaded box entails hematological tumors. (Tissue boxplot from http://ist.medisapiens.com/).

Preferentially Expressed Antigen in Melanoma (PRAME) is a cancer/testis antigen initially isolated from melanoma cells [1]. *PRAME* has been shown to be overexpressed in an array of solid and hematological malignancies [1, 2]. Expression in healthy tissues is mainly restricted to testis, but low levels have also been found in the endometrium, ovaries and adrenal glands [1]. In hematological malignancies, *PRAME* has displayed particularly high expression in Acute Myeloid Leukemia (AML), Acute Lymphoid Leukemia (ALL) and Chronic Myeloid Leukemia (CML) in blast crisis [2, 3] (Figure 1). Overexpression of PRAME is also common in solid malignancies, particularly melanoma and nephroblastoma (Figure 1).

Although the pathophysiological function of PRAME remains unclear, considerable work to understand the effect of its re-emergence in progressive disease is underway. There is evidence indicating that PRAME could be a repressor of the Retinoic Acid Receptor (RAR) and thereby antagonize the antiproliferative and cytotoxic effects of Retinoic Acid (RA) [4]. Its predictive capacity as a disease biomarker is complex and seems to vary between malignancies. In solid tumors, aberrant PRAME expression has been associated with poor prognosis [5, 6], while it has been found to predict a more favorable outcome in AML [7]. In CML, PRAME expression is higher in blast phase, suggesting a role in disease progression [8, 9]. Across disease types, PRAME might be useful for monitoring of minimal residual disease in various hematological malignancies [3].

Proto-Siqueira *et al.* previously reported that PRAME, apart from being associated with cytoplasmic and nuclear-associated protein, was detectable as a membrane protein [10]. The combination of the expression profile and cancer biomarker properties of PRAME encouraged us to develop an antibody (MPA1) specific for what could theoretically be an extracellular epitope of PRAME. Here, we have applied this antibody for diagnostic purposes *in vitro* using flow cytometry and high magnification confocal microscopy. To investigate the *in vivo* targeting properties of MPA1, we constructed an immuno-PET radiotracer by labeling the antibody with zirconium-89, a positron-emitting radioisotope with a 3.3 day half-life, which is well suited for studying antibodies *in vivo*. Our results demonstrate that a PRAME targeting approach has considerable potential to effect targeted imaging and therapy of multiple cancers.

## RESULTS

### Specific PRAME binding of MPA1 by ELISA

**Figure 2:**
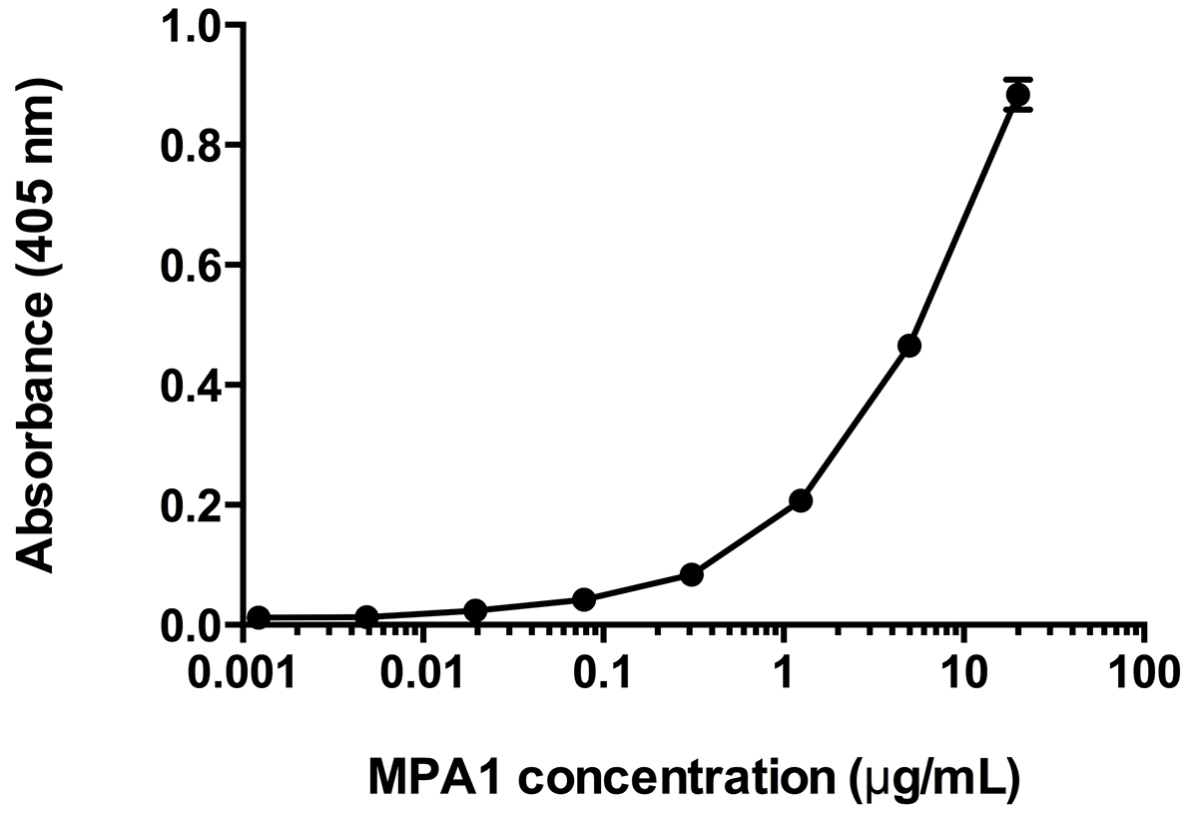
Enzyme-Linked Immunosorbent Assay (ELISA) assessment of MPA1 biding to PRAME-Fc fusion protein MPA1 was serially diluted and added to wells containing immobilized PRAME-Fc fusion protein. An AP-conjugated secondary antibody allowed detection and absorbance corresponds to binding. Data is expressed as mean of duplicate results.

To determine if MPA1, developed against a domain of PRAME, binds to PRAME efficiently, we performed an ELISA. The results showed specific binding of MPA1 to a PRAME-Fc fusion protein consisting of amino acids 23–407 (Figure 2).

### PRAME expression and cellular localization

**Figure 3:**
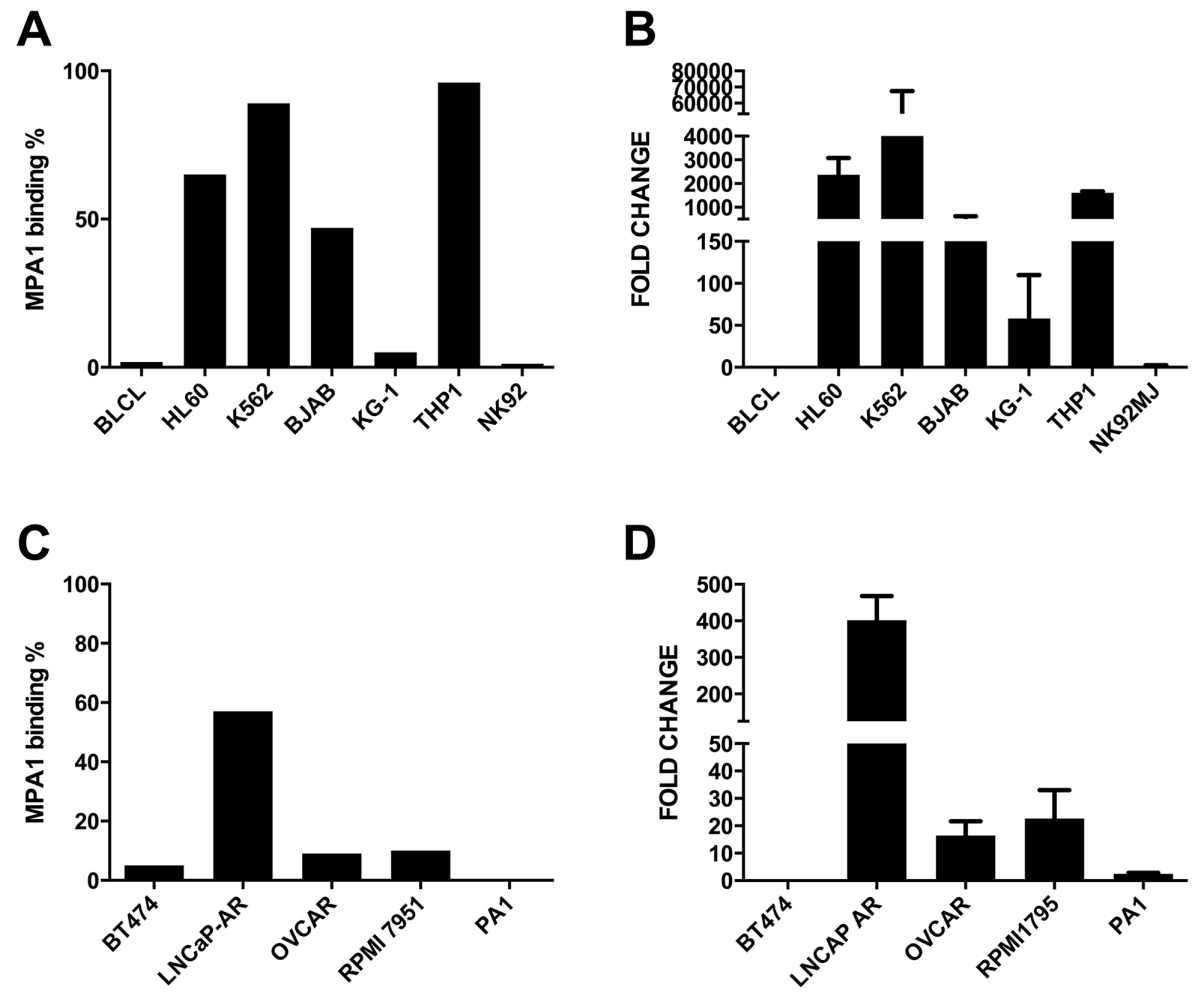
MPA1 cell surface staining of liquid **(A)** and solid **(C)** tumors by flow cytometry; PRAME mRNA expression by RT-PCR **(B)** for liquid tumors in comparison to BLCLs and **(D)** for solid tumors in comparison to BT474 cells.

We studied MPA1 binding by flow cytometry in both solid (OVCAR, PA1, RPMI-7951, BT474, LNCaP-AR) and liquid (BLCL, HL60, K562, BJAB, KG-1, THP1, NK92) tumor cell lines using an extracellular staining protocol (Figure 3A and 3C). To compare surface staining with *PRAME* gene expression, RT-PCR was performed on all cell lines (Figure 3B and 3D). mRNA expression was normalized to the cell line with the lowest *PRAME* expression in each group; BLCL and BT474 among liquid and solid tumors, respectively. mRNA expression largely correlated with cell membrane staining.

**Figure 4:**
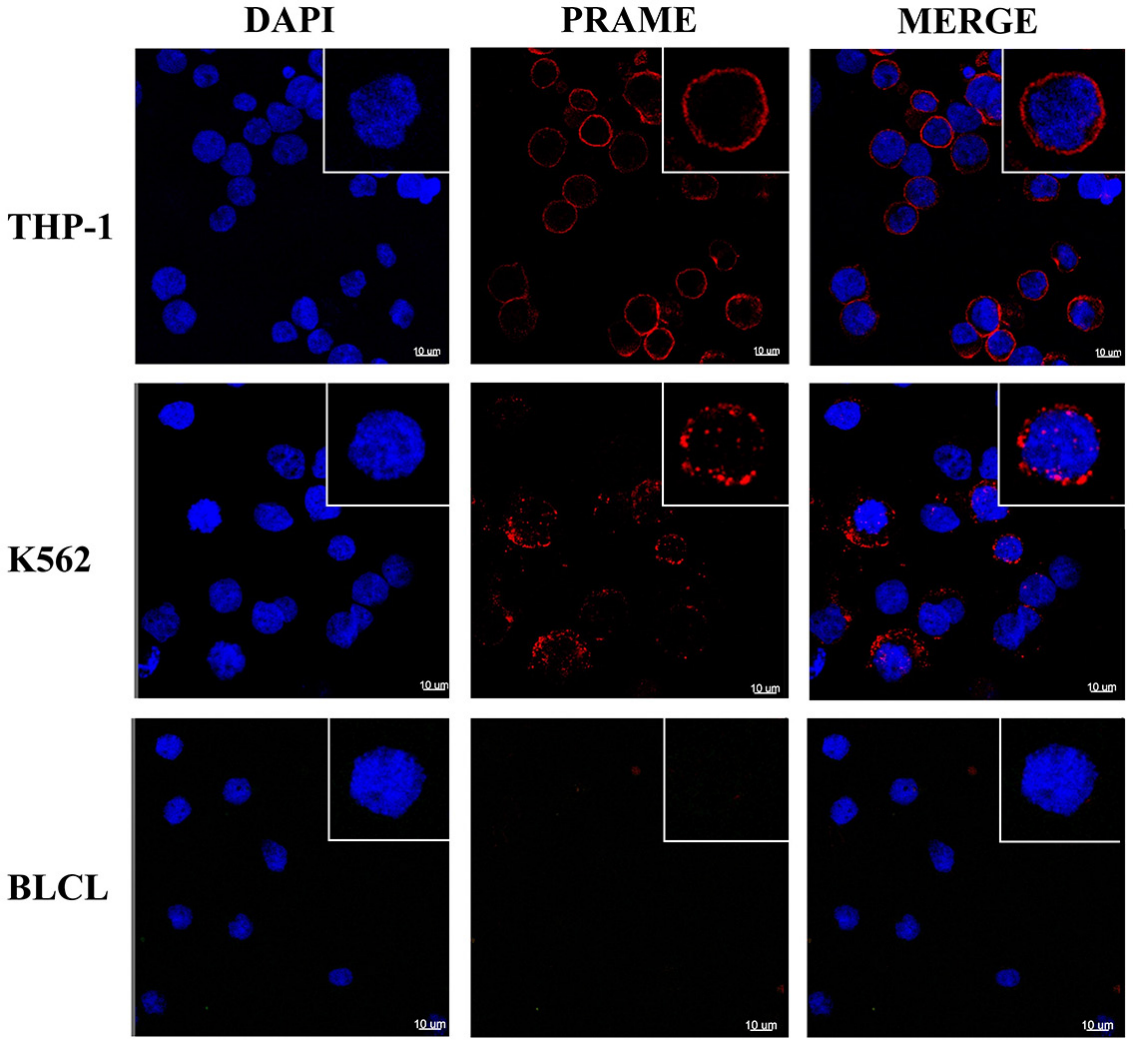
PRAME staining of cellular membrane using confocal microscopy Representative images showing intense membrane-associated staining of MPA1 (red channel) in PRAME positive THP-1 and K562 cells but not in PRAME negative BLCL. Staining with DAPI (blue channel) displays cell nuclei. Single cells in magnification are shown in the upper right corners.

Cellular localization of PRAME by immuno-fluorescence microscopy showed binding of the MPA1 antibody to the cellular membrane of K562 and THP-1 (PRAME positive), while BLCL (PRAME negative) showed no binding (Figure 4). Median Fluorescent Intensity (MFI) of PRAME was measured at different stages of the cell cycle (G/0, S-phase, and G2/M), which we determined by DNA levels. We found no correlation between PRAME staining and a specific phase of the cell cycle (data not shown).

To further evaluate the specificity of MPA1, we investigated if staining increased with PRAME induction. Inhibition of DNA methylation has been shown to augment *PRAME* in AML cell lines [9, 11, 12, 13]. MPA1 staining of HL60, cultured in 5-Azacytidine (5-AZA) containing cell media (0.05 nM/ml 5-AZA, Sigma), increased by 20% and 10%, after 24 and 48 hours, respectively, compared to untreated cells.

### ^89^Zr-DFO-MPA1 as a probe for PRAME expression

**Figure 5:**
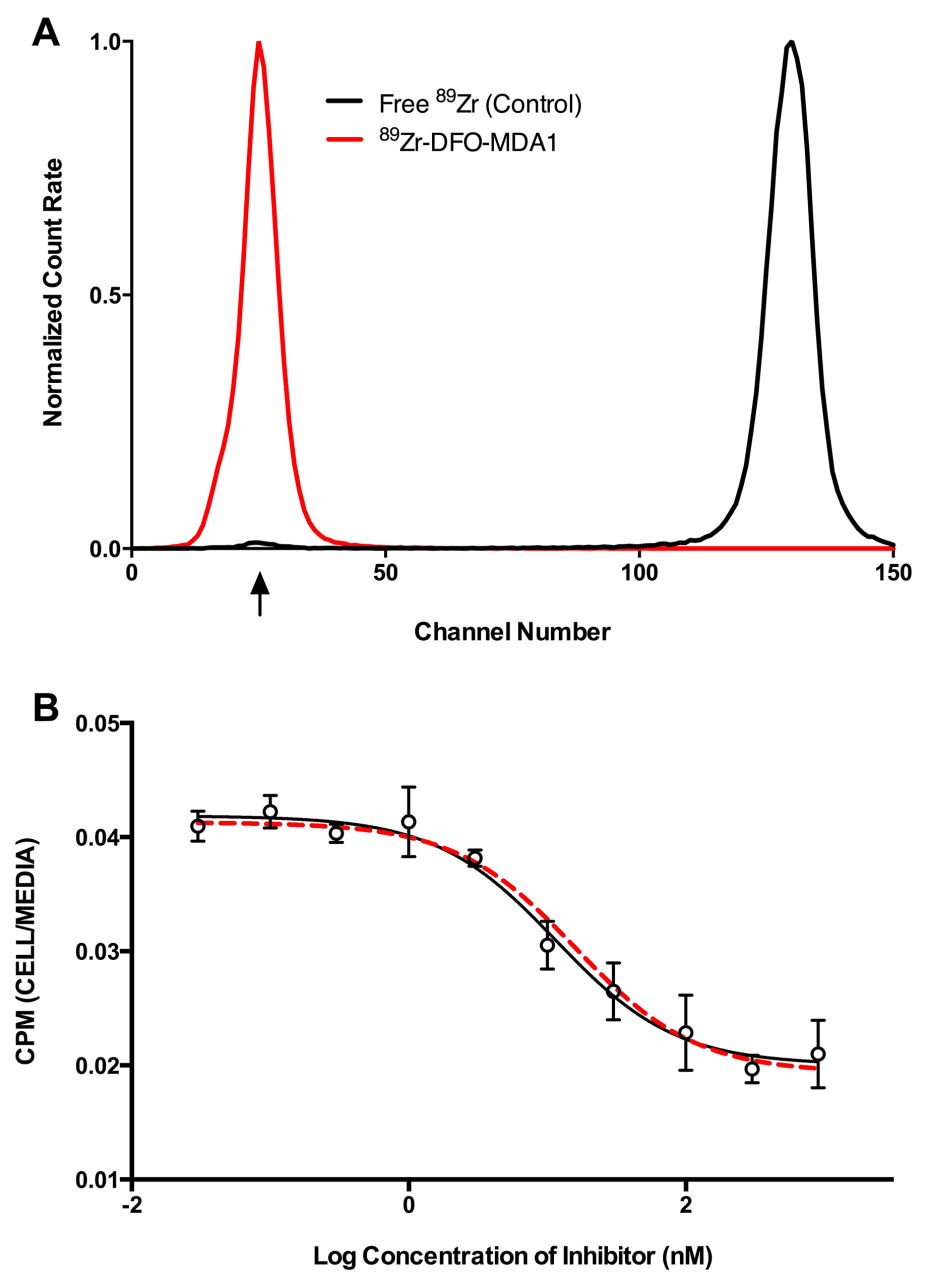
*In vitro* characteristics of ^89^Zr-DFO-MPA1 **(A)** Representative radio-ITLC (instant thin layer chromatography) of ^89^Zr-DFO-MPA1 showing high radiochemical purity (>99%) as ^89^Zr-DFO-MPA1 remains at baseline and free ^89^Zr was undetectable (developed in 50mM DTPA). **(B)** Competitive binding assay of ^89^Zr-DFO-MPA1 in THP-1 cells indicate no significant loss of affinity during radiolabeling. Radiotracer binding gradually decreases with incremental increase of unlabeled MPA1 blocking (IC_50_=11.51 nM).

In order to assess MPA1-PRAME binding in animal models of cancer, we constructed a positron-emitting radionuclide (zirconium-89) radiolabeled antibody conjugate for noninvasive imaging. Radiopurity was assessed by radio-instant thin layer chromatography. Briefly, ^89^Zr-DFO-MPA1 was blotted (1 μL) on silica-impregnated paper and a solution of 50 mM diethylenetriaminepentaacetic acid was used as a mobile phase (Figure 5A). All labeling reactions achieved >99% radiochemical purity. We observed an IC_50_ of 11.5 nM (R^2^=0.952) for ^89^Zr-DFO-MPA1 in a competitive binding assay with unlabeled MPA1 in THP-1 cells (Figure 5B), demonstrating that the radiolabeled construct retained high affinity and specificity.

### Biodistribution and PET imaging of ^89^Zr-DFO-MPA1 in tumor models

**Figure 6:**
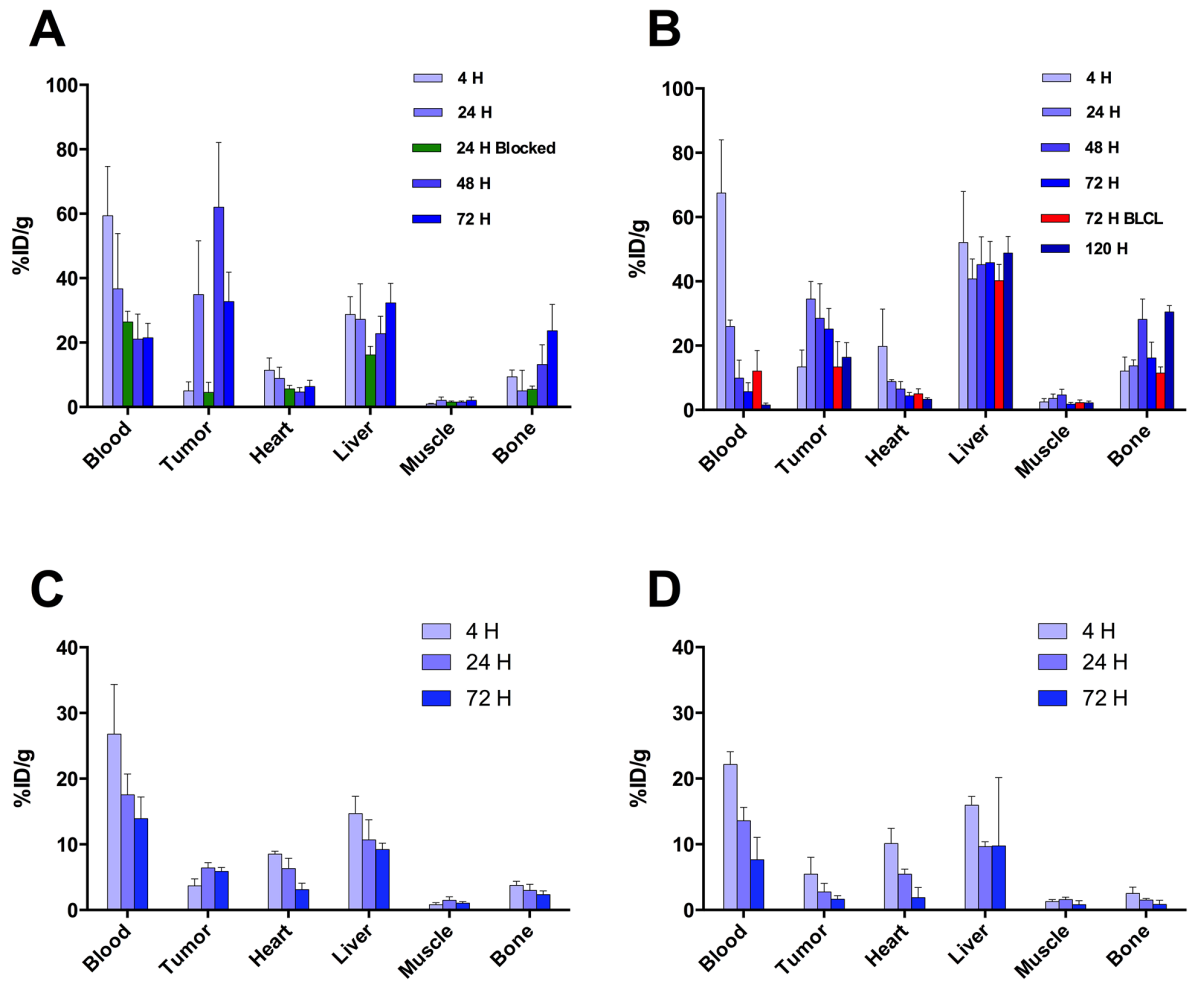
Biodistribution of ^89^Zr-DFO-MPA1 in selected tissues of mice xenografts **(A)** Biodistribution in mice bearing bilateral K562 xenografts, examined at multiple time points (n = 4-5 per group) showing peak tumor uptake at 24 hours. **(B)** Significantly (p<0.01) lower uptake of ^89^Zr-DFO-MPA1 was noted in PRAME negative xenograft BLCL (n = 4) at 72 hours. **(C)** Biodistribution in mice bearing bilateral THP-1 xenografts. Tissue uptake revealed a similar pattern, with peak tumor uptake at 48 hours. A separate blocking group (n=4) at 24 hours were co-administered tracer with unlabeled MPA1 that significantly (p<0.01) decreased tumor uptake. Data is expressed as mean %IA/g (± one standard deviation). **(D/E)** Biodistribution in mice bearing bilateral LNCaP-AR s.c. xenografts, a PRAME positive solid tumor, showed significantly (P < 0.01) higher ^89^Zr-DFO-MPA1 tumor uptake than BT474 s.c. xenografts, a PRAME negative solid tumor.

To utilize this novel imaging probe *in vivo* in order to characterize cell surface expression of PRAME in multiple cancer models we performed classic organ biodistribution assays. Maximum tumor-associated activity of ^89^Zr-DFO-MPA1 was observed 24 hours post injection (p.i.) in K562 models (34.5 ± 5.5 %IA/g, tumor-to-muscle ratio: 9.6) and 48 hours p.i. for THP-1 (62.0 ± 20.1 %IA/g, tumor-to-muscle ratio: 40.1), respectively (Figure 6). Co-injection of a blocking dose of unlabeled MPA1 significantly (P<0.01) decreased radiotracer uptake in THP-1 xenografts (Figure 6B; Table 1B). Uptake of ^89^Zr-DFO-MPA1 was significantly lower (P<0.01) in PRAME-negative BLCL compared to PRAME positive liquid tumor models (Table 1A). Similarly, a PRAME negative solid tumor model (BT474 breast carcinoma) exhibited a significantly lower uptake of ^89^Zr-DFO-MPA1 than the PRAME positive LNCaP-AR prostate cancer xenograft (Figure 6C and 6D; Table 2A and 2B). Generally, off-target ^89^Zr-DFO-MPA1 accumulation was minimal in host tissues, with the exception of spleen uptake in K562 xenografts (917.4 ± 417.9 %IA/g at 48 hours p.i.). We did not note a high spleen uptake in BALB/c (nu/nu) based models (BT474, LNCaP-AR), which also showed a lower uptake in general.

**Table 1:** Full ex vivo biodistribution data of ^89^Zr-DFO-MPA1 over time (hours, h) in PRAME positive K562 and negative BLCL (A) and THP-1 (B) tumor bearing male NSG mice. Biodistribution in PRAME negative BLCL tumor bearing mice is displayed at 72 h **(A).** At 24 h in THP-1 **(B)** a group of mice (n = 4)were co-administered with unlabeled MPA1 to block uptake. Data is expressed as mean %IA/g (± one standard deviation)

A. K562						
	4 h	24 h	48 h	72 h	72 h BLCL	120 h
Blood	67.5 ± 16.5	26.0 ± 1.9	9.9 ± 5.6	5.7 ± 2.8	12.1 ± 6.4	1.5 ± 0.7
Tumor	13.4 ± 5.2	34.5 ± 5.5	28.5 ± 10.8	25.2 ± 6.3	13.4 ± 7.9	16.4 ± 4.5
Heart	19.8 ± 11.6	8.8 ± 0.6	6.5 ± 2.3	4.4 ± 1.0	5.0 ± 1.6	3.3 ± 0.5
Lungs	44.9 ± 15.2	20.8 ± 1.9	10.9 ± 2.9	7.0 ± 2.0	7.5 ± 3.2	4.4 ± 0.6
Liver	52.1 ± 15.9	40.8 ± 6.2	45.2 ± 8.7	45.8 ± 6.6	40.2 ± 5.1	48.8 ± 5.2
Spleen	51.4 ± 25.6	162.7 ± 33.4	917.4 ± 417.9	248.4 ± 78.5	40.0 ± 4.4	328.2 ± 39.9
Stomach	3.2 ± 1.6	6.3 ± 2.3	4.9 ± 0.5	3.5 ± 0.6	3.6 ± 2.0	2.1 ± 0.4
Sm. Intestine	5.7 ± 3.4	11.6 ± 1.8	12.9 ± 1.8	9.2 ± 1.5	11.1 ± 2.7	7.9 ± 2.0
Lg. Intestine	9.8 ± 4.1	8.3 ± 1.1	9.8 ± 1.9	7.0 ± 1.3	5.1 ± 1.0	4.3 ± 0.5
Kidneys	20.4 ± 8.6	11.8 ± 0.3	8.9 ± 1.2	7.8 ± 1.6	7.3 ± 1.7	6.4 ± 0.7
Muscle	2.5 ± 1.0	3.6 ± 1.4	4.7 ± 1.7	1.8 ± 0.5	2.3 ± 0.8	2.2 ± 0.6
Bone	12.1 ± 4.4	13.7 ± 1.9	28.2 ± 6.3	16.2 ± 4.9	11.5 ± 1.9	30.5 ± 2.0
Tail	13.2 ± 10.6	9.5 ± 4.0	9.4 ± 5.6	5.6 ± 1.4	5.9 ± 1.2	5.7 ± 1.0
Testis	10.5 ± 5.3	6.0 ± 0.6	5.4 ± 0.8	5.3 ± 0.3	5.9 ± 1.0	5.1 ± 0.3

B. THP-1					
Organ	4 H	24 H	24 H Block	48 H	72 H
Blood	59.4 ± 15.3	36.7 ± 17.2	26.4 ± 3.3	21.1 ± 7.8	21.5 ± 4.5
Tumor	5.0 ± 2.8	34.9 ± 16.9	4.5 ± 3.2	62.0 ± 20.1	32.7 ± 9.2
Heart	11.4 ± 3.8	8.9 ± 3.5	5.6 ± 1.1	4.7 ± 1.3	6.4 ± 1.8
Lungs	47.7 ± 20.1	21.9 ± 8.6	16.5 ± 1.8	13.1 ± 5.7	10.7 ± 3.3
Liver	28.7 ± 5.5	27.2 ± 11.1	16.2 ± 2.6	22.8 ± 5.3	32.3 ± 6.1
Spleen	23.8 ± 4.7	41.9 ± 13.8	10.4 ± 2.1	54.0 ± 24.5	70.3 ± 14.2
Stomach	2.5 ± 0.6	3.7 ± 1.1	1.7 ± 0.1	2.7 ± 0.4	4.2 ± 1.0
Sm. Intestine	5.1 ± 0.7	8.0 ± 2.2	2.6 ± 0.3	7.4 ± 1.5	10.7 ± 1.6
Lg. Intestine	5.3 ± 0.5	5.6 ± 1.3	2.5 ± 0.2	4.4 ± 1.1	6.0 ± 1.4
Kidneys	13.8 ± 2.8	10.9 ± 4.4	6.4 ± 0.7	7.5 ± 2.4	8.1 ± 1.1
Muscle	0.9 ± 0.2	2.1 ± 1.0	1.5 ± 0.4	1.5 ± 0.4	2.1 ± 1.0
Bone	9.4 ± 2.1	5.0 ± 6.4	5.5 ± 1.0	13.2 ± 6.1	23.7 ± 8.2
Tail	16.1 ± 10.2	5.2 ± 2.3	6.4 ± 3.7	6.1 ± 1.6	6.1 ± 3.1
Testis	6.0 ± 1.2	9.6 ± 5.8	3.2 ± 0.3	4.7 ± 0.8	5.6 ± 0.6

**Table 2:** Full ex vivo biodistribution data of ^89^Zr-DFO-MPA1 over time (hours, h) in PRAME negative **BT474 (A)** and positive **LNCaP-AR (B)** tumor bearing male BALB/c mice

A. BT474			
	4 h	24 h	72 h
Blood	22.2 ± 1.9	13.6 ± 2.0	7.6 ± 3.4
Tumor	5.4 ± 2.6	2.8 ± 1.3	1.7 ± 0.5
Heart	10.1 ± 2.3	5.5 ± 0.8	1.9 ± 1.5
Lungs	13.1 ± 5.3	7.9 ± 1.6	3.3 ± 3.6
Liver	15.9 ± 1.4	9.7 ± 0.7	9.8 ± 10.4
Spleen	7.4 ± 6.1	5.3 ± 0.9	1.9 ± 1.6
Stomach	1.5 ± 1.2	1.4 ± 0.1	0.6 ± 0.4
Sm. Intestine	2.9 ± 0.6	2.2 ± 0.4	0.7 ± 0.4
Lg. Intestine	4.5 ± 2.1	1.8 ± 0.2	2.1 ± 1.9
Kidneys	6.9 ± 3.6	5.5 ± 0.5	3.9 ± 3.0
Muscle	1.3 ± 0.3	1.6 ± 0.4	0.8 ± 0.6
Bone	2.5 ± 0.9	1.5 ± 0.2	0.8 ± 0.6
Tail	4.2 ± 0.6	4.7 ± 3.6	1.2 ± 0.2

B. LNCaP-AR			
	4 h	24 h	72 h
Blood	26.8 ± 7.6	17.6 ± 3.1	13.9 ± 3.3
Tumor	3.7 ± 1.1	6.4 ± 0.8	5.9 ± 0.6
Heart	8.5 ± 0.4	6.3 ± 1.6	3.1 ± 1.0
Lungs	11.1 ± 2.4	8.8 ± 1.8	5.1 ± 1.3
Liver	14.7 ± 2.6	10.7 ± 3.1	9.2 ± 1.0
Spleen	14.1 ± 1.5	10.0 ± 2.4	7.4 ± 1.5
Stomach	1.1 ± 0.4	1.2 ± 0.2	0.8 ± 0.4
Sm. Intestine	2.7 ± 0.5	2.7 ± 0.5	1.1 ± 0.2
Lg. Intestine	2.2 ± 0.5	1.7 ± 0.2	0.8 ± 0.1
Kidneys	8.7 ± 2.4	6.5 ± 1.2	4.9 ± 0.5
Muscle	0.8 ± 0.3	1.5 ± 0.6	1.1 ± 0.2
Bone	3.8 ± 0.6	3.0 ± 0.9	2.3 ± 0.6
Tail	5.5 ± 3.6	3.2 ± 0.8	2.8 ± 0.4
Testis	3.7 ± 0.4	3.6 ± 0.5	3.1 ± 0.5

**Figure 7:**
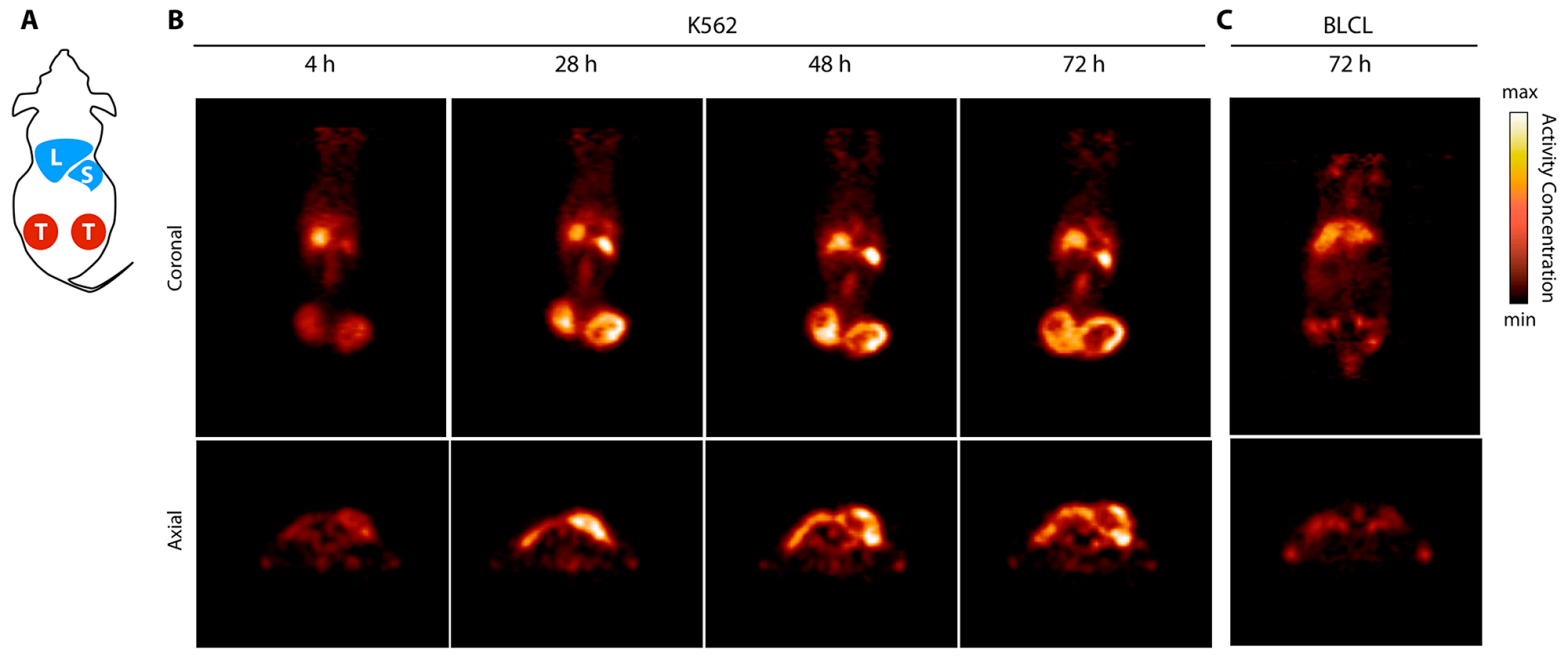
ImmunoPET imaging of PRAME positive tumors using ^89^Zr-DFO-MPA1 **(A)** Schematic cartoon of a s.c. tumor model displaying liver (L), spleen (S) and bilateral tumors (T). **(B)** Coronal and axial PET slices over time (hours, H) in a representative PRAME positive K562 xenograft bearing mouse, with addition of a PRAME negative BLCL xenograft bearing mouse at 72 h. Delineation of the K562 from background is clear due to the high tumor-to-background contrast. **(C)** In comparison, the BLCL tumors which do not express the extracellular PRAME binding epitope are not visualized by ^89^Zr-DFO-MPA1.

The ^89^Zr-DFO-MPA1 construct also enables noninvasive longitudinal evaluation through positron emission tomography (PET) imaging. In the K562 xenograft model, we evaluated the organ pharmacokinetics and tumor uptake over the course of 72 h. The studies showed a region of focal uptake in K562 tumors, recapitulating the biodistribution data (Figure 7), enabling clear delineation of the malignant mass.

## DISCUSSION

PRAME is among of the most widely studied cancer/testis antigens and has been proposed as a promising cancer biomarker and therapeutic target. The prevailing view of PRAME has been as that it is exclusively an intracellular protein and because of this, has been largely overlooked as a target for imaging agents and macromolecular therapeutics. Innovative immunotherapy concepts to circumvent this issue have previously been suggested. Therapeutic cancer vaccines consisting of PRAME peptides have entered clinical trials for patients with different types of solid tumors and have not shown any major toxicities [14, 15, 16]. Although the vaccinal approach to treat cancer is attractive from a methodological perspective, treatment efficacies have generally been low. In fact, early trial results of the effect of a PRAME vaccine in patients with lung cancer have been underwhelming. The putative lack of significant therapeutic effect has prompted a halt in further development of immunological approaches to target PRAME [14].

In this work, we demonstrate, in multiple tumor models, that PRAME can be targeted *in vivo* by antibodies specific for an extracellular domain. Our results independently confirm previous results demonstrating that PRAME is associated with the cell membrane. These findings may provide an additional approach to PRAME targeting using molecularly specific targeting agents. We constructed an antibody (MPA1) against amino acid region 310-331 of PRAME, which we predicted to be extracellular. Flow cytometry showed binding of MPA1 to PRAME positive, but not to PRAME negative cell lines, ensuring MPA1 target specificity *in vitro*. To abrogate binding to intracellular PRAME, we used an extracellular staining protocol, without a permeabilization step to prevent MPA1 from interfering prior to cell fixation.

In comparison to PRAME mRNA levels, flow cytometry of MPA1 on THP-1 was disproportionately higher than in K562. However, disproportionality between transcription and mature protein is a commonly occurring biological phenomenon [17, 18, 19]. It has previously been suggested that the distribution of PRAME between the nuclear and cytoplasmic compartments varies in different cell types and conditions [20] and we suggest a similar model with the addition of a third cellular localization site (i.e. cell membrane) of PRAME. This assists in explaining the difference in expressed cell surface PRAME from mRNA, exhibited by these cell lines. Post-transcriptional defects, misfolding, and many other factors could result in such discrepancies [21, 22]. Surface binding was further confirmed by the outcome of our confocal microscopy studies, which clearly visualized that MPA1 staining was localized to the cellular membrane of PRAME positive cell lines THP-1 and K562, but not to PRAME negative BLCL.

We showed that MPA1 binding increases significantly after PRAME expressing cells were treated with 5-AZA, which is known to induce PRAME expression. This demonstrates that membrane bound PRAME is representative of intra-cellular PRAME, and that MPA1 can be used to report changes in PRAME expression levels following pharmacological stimulation. To further our understanding of the biology of PRAME, we investigated if expression of membrane bound PRAME was correlated to certain phases of the cell cycle. Our results showed that PRAME was constituently expressed on the cell surface and not associated with a specific cell cycle phase. This increases the possibility of successful development of immunotheranostic targeting of membrane bound PRAME.

In order to study and evaluate PRAME as a cancer specific target with translational capability, we synthesized a radiolabeled MPA1. Both *in vivo* immuno-PET and *ex vivo* biodistribution showed accumulation of ^89^Zr-DFO-MPA1 that was significantly higher in PRAME positive, compared to PRAME negative, mouse models of disease. Binding specificity was further validated by our blocking study in which unlabeled antibody successfully blocked tumor uptake of ^89^Zr-DFO-MPA1 in THP-1 xenograft. Off-target uptake was mainly restricted to liver, spleen and bone, while blood-bearing organs such as heart and lungs showed a decrease rate matching blood clearance. The non-decreasing liver uptake is likely a result of the well-known fact of immunoglobulin retention and processing in the liver, and the increasing bone uptake is likely caused by free ^89^Zr^4+^ ion, which has high affinity for bone, released from metabolic degradation of the tracer [23]. Translational studies have shown that compared to preclinical models, ^89^Zr^4+^ bone deposition is lower in humans [24].

Unexpectedly, we noted that all three NSG based models of liquid tumors had a high spleen uptake. In the K562 tumor model, the higher spleen uptake is most likely associated with the nature of the malignancy and has been previously described in this model [25]. However, it should be noted that the high uptake was associated with very low organ weight (data not shown). Additionally, the splenic uptake could be blocked with unlabeled MPA1, indicating a specific affinity. Whether this specific binding is caused by splenic migration of tumor cells or by cross-reactivity to an epitope similar to PRAME was not confirmed in this study. Splenic uptake could also be related to host immunity [26]; significant uptake was only noted in murine NSG SCID based liquid tumor models, as opposed to solid. Also, the use of a polyclonal antibody increases the risk of cross-reactivity, and binding to healthy splenic cells will be monitored carefully while selecting a lead monoclonal antibody candidate for clinical translation.

With these encouraging proof-of-principle results in hand, we have begun to study applications of PRAME targeting. We are currently generating monoclonal antibodies that target the same extracellular epitope domain that MPA1 targets; in residues 310–331 of PRAME. These monoclonals would then be ideally suited for examining therapeutic potential and off-target binding, with the overarching long-term goal to develop a therapeutic selective for PRAME positive cancers. Since many malignancies overexpress PRAME, the potential applications are manifold, from diagnostic imaging and therapy to treatment guidance.

## MATERIALS AND METHODS

### Development of a polyclonal antibody

Computational transmembrane analysis of PRAME predicted that amino acid region 310-331 could be extracellular. A polyclonal IgG rabbit antibody, denominated MPA1 was obtained in collaboration with Innovagen AB, Lund, Sweden. A peptide consisting of this 22 amino acid long sequence was produced, conjugated to Keyhole Limpet Hemocyanin (KLH), and used as immunogen in rabbits. Antibodies were purified from serum by Protein G chromatography followed by affinity purification with the peptide previously used as immunogen.

### Indirect Enzyme-Linked Immunosorbent Assay (ELISA)

A Nunc Maxisorp 96 MicroWell plate (Thermo Fisher Scientific, Waltham, MA, USA) was coated with a PRAME-Fc fusion protein consisting of amino acids 23-407 (Innovagen AB, Lund, Sweden), 1 μg/ml in PBS, at 4° C overnight. The plate was then washed with PBS-Tween 0.05% and blocking solution (PBS, 0.5% BSA, Tween 20, pH 7.4), was added to each well. The plate was covered and placed on a shaker for 1 hour at room temperature, and then washed again. MPA1 was serially diluted from 20 μg/mL to 0.00122 μg/mL and added to the plate, followed by shaking incubation for 1 hour at room temperature. A goat-anti rabbit antibody conjugated to alkaline phosphatase (Agilent Technologies, Santa Clara, CA, USA) was used as secondary antibody for detection. The antibody was diluted 1:2000 in the blocking solution and incubated in the wells for 1 hour at room temperature, preceded and followed by washing of the plate. *Para*-Nitrophenylphosphate (pNPP) was used as detection substrate and added at 1 mg/mL in pNPP-buffer (0.2 M Tris, 1 mM MgCl, pH 9.8). The plate was read immediately for background optical absorbance and after incubation, at 405 nm in a Multiskan EX microplate spectrophotometer (Thermo Fisher Scientific, Waltham, MA, USA).

### Cell lines

HL60, THP-1, K562, NK92, KG-1, BT474, OVCAR, RPMI1795, and PA1 were purchased from American Type Culture Collection (ATCC, Manassas, VA, USA). LNCaP-AR was a kind gift from the Sawyers laboratory. BJAB was kindly provided by Scheinberg laboratory. K562 is a CML cell line in blast crisis and THP-1, HL60 and KG-1 are AML cell lines. BJAB and NK92 are lymphoma cell lines, Burkitt and non-Hodgkin, respectively. OVCAR and PA-1 are ovarian cancer cell lines, while RPMI-7951 and LNCaP-AR are lymph node metastases of melanoma and prostate cancer, respectively. BT474 is a ductal breast carcinoma. All cell lines were cultured according to the manufacturer’s instructions. 5-Azacytidine (Sigma), 0.05 nM/ml 5-AZA, was added to the cell media to inhibit of DNA methylation. Epstein-Barr virus (EBV)-transformed B-lymphoblastoid cell line (BLCL) was obtained through transformation with EBV virus of healthy donor B-cells collected by Ficoll density centrifugation. The donor cells were collected after informed consent according to Memorial Sloan Kettering Cancer Center Institutional Review Board.

### Flow cytometry

Approximately 0.5 × 10^6^ cells per cell line were harvested, washed in phosphate-buffered saline (PBS) and incubated with MPA1 (10 μg/mL) for 20 minutes at 4° C. Alexa 488 Goat anti-Rabbit polyclonal IgG (Abcam Biotechnology) was used as secondary antibody and cells were stained for 15 minutes at 4° C (10 μg/mL). Flow cytometry data was collected on a BD FACSCalibur (BD Biosciences, San Jose, CA, USA) and analyzed with FlowJo 9.4.8 software. When investigating correlations between PRAME expression (by quantifying MPA1 binding) and cell cycle phases, cells were washed and fixed with 4% PFA (Thermo Scientific) for 15 minutes at room temperature (RT), spun down for 5 minutes, and permeabilized with PBS with 0.5% Triton X-100 (Fisher Scientific) at RT for 15 min, followed by two washes before adding DAPI (4’,6-diamidino-2-phenylindole). Samples were acquired on a LSR Fortessa (BD Biosciences, San Jose, CA, USA) and analyzed with FCS Express 6 software.

### Reverse transcription polymerase chain reaction (RT-PCR)

2 × 10^6^ cells per cell line were harvested, pelleted and frozen prior to the assay. RNA was extracted using the RNeasy Mini Kit (250) (Qiagen, Hilden, Germany) and cDNA was generated using High-Capacity cDNA Reverse Transcription Kit (Applied Biosystems, Foster City, CA, USA), both according to the manufacturers’ instructions. PRAME specific primers were obtained from Qiagen and DNA amplification was performed using the RT2 SYBR Green fluor qPCR Mastermix (Qiagen, Hilden, Germany) and the CFX96 Touch™ Real-Time PCR Detection System (Bio-Rad Laboratories Inc., Hercules, CA, USA). All data was obtained in triplicate and the mRNA expression levels were analyzed and normalized to HTB78, BLCLs and BT474 cells, (a cell lines with very low PRAME expression), and presented as fold change.

### Indirect immunofluorescence microscopy

Cells were transferred from culture to 15 mL centrifuge tubes (BD Biosciences, San Jose, CA, USA) and washed three times with cold phosphate-buffered saline (PBS). The pellets were resuspended in 100 μL of cold PBS and put on ice. The cells were incubated with MPA1 (70 μg/mL) for 90 minutes, kept on ice to prevent internalization, followed by three washes with PBS and pellet resuspension to a concentration of 1 × 10^6^ cells/mL in PBS. Cells were transferred to 4 well chamber slides, 2 × 10^5^ cells per well. The slides were centrifuged at 1000 RPM and 4° C for 5 minutes. 200 μL of 4% paraformaldehyde was added to each well, already containing 200 μL of PBS. The slides were centrifuged again for 2 minutes (1000 RPM and 4° C). The supernatants were carefully poured off and 500 μL of PBS were added and removed by pipetting. The chambers were removed and the slides were dipped in PBS and allowed to air-dry followed by oven drying at 60° C for 30 minutes. The chambers were put back on and the fixed cells were incubated with goat Anti-Rabbit IgG Alexa 568 (Invitrogen, Carlsbad, CA, USA; 1:500 dilution) in darkness at room temperature for 45 minutes, followed by three washes with PBS. Chambers were removed and coverslips were mounted using Vectashield Hardset Mounting Media with DAPI (Vector Laboratories, CA, USA).

Confocal laser scanning microscopy was performed using the Leica TCS SP8 X confocal microscope (Leica Microsystems GmbH, Wetzlar, Germany) in the Molecular Cytology Core Facility at MSKCC. Images were rendered using Fiji and Imaris software.

### Preparation of ^89^Zr-DFO-MPA1

MPA1 was used as received from Innovagen AB, Lund, Sweden. Buffers and reagents used prior to and during radiolabeling were treated with metal scavenging resin at 5% wt/vol overnight (BT Chelex 100 resin, Bio-Rad Inc., Hercules, CA, USA). Protein concentrations were determined by UV-vis spectroscopy (NanoDrop 2000). MPA1 was buffer-exchanged four times in pH 8.5 0.5M HEPES using a 50 kDa cutoff spin filter (5,000×g, 2° C, Amicon Ultra-4, Merck-Millipore, Cork, Ireland). MPA1 was concentrated to a volume of 200μL, transferred and an additional 100μL of buffer was used to rinse the membrane. MPA1 was then derivatized with *para*-isothiocyanatobenzyl DFO (B-705, Macrocyclics, Plano, TX, USA) as follows. To the buffer exchanged antibody (3 mg in ∼300 μL, 20 nmol) was added three aliquots of 5 μL *p*-SCN-Bn-DFO (10mg/ml in DMSO, 200 nmol, 10-fold excess) with brief, gentle vortexing after each addition; this was incubated for 45 min at 37° C. The DFO-conjugated MPA1 was buffer exchanged into pH 7.5 0.5 M HEPES four times in an Amicon spin-filter in the same manner as above. The total IgG concentration was measured before and after DFO conjugation with a yield typically exceeding 80%. The DFO conjugate could be stored at this point, and was typically used in <24h.

Zirconium-89 (^89^Zr) in 1 M oxalic acid was used as supplied in a microfuge tube (RMIP Core, MSKCC). The ^89^Zr stock was neutralized by adding small aliquots of 1M Na_2_CO_3_ until neutral by narrow range pH paper. After each addition of base, the solution was mixed gently by pipetting; after completion, the tube was left a few minutes to allow oxalate salts to precipitate. This was transferred to a microfuge tube filter (Costar 8169, Corning, NY), and the salts were removed at low-speed for 1-2min in a minifuge. The clear ^89^Zr solution was added to the DFO-MPA1 conjugate, mixed gently by pipetting and incubated at room temperature for 1 hour. Radio-instant thin-layer chromatography (Radio-ITLC) in 50mM DTPA was performed to confirm consumption of free ^89^Zr. DTPA (20μL, 50mM) was added to the reaction mixture to chelate any remaining free Zr-89. Crude ^89^Zr-DFO-MPA1 was purified by size exclusion chromatography on a PD-10 column (GE Healthcare, Buckinghamshire, UK). The column was equilibrated with 20 mL of sterile 0.9% saline by gravity elution. The labeling reaction was loaded to the column in its entirety, typically in a volume of 600-800 μL. The ^89^Zr-DFO-MPA1 was eluted with saline in 0.5 mL fractions, pooled and evaluated by radio-ITLC for radiochemical purity. The radiolabeling yield ranged from 75.5 to 87.5% with a final specific activity between 1.36 and 2.59 mCi/mg of antibody. The mass of ^89^Zr-DFO-MPA1 was assumed to be proportional to radioactivity for yield and specific activity calculations.

### Competitive binding assay

Unlabeled (non-radioactive) MPA1 antibody was serially diluted in RPMI 1640 with 1% BSA, from 900 nM to 0.03 nM in triplicate set of vials. The unlabeled antibody dilutions were transferred to vials containing 1 × 10^6^ THP-1 cells each. ^89^Zr-DFO-MPA1 (1 μCi) in 50 μl of RPMI 1640, 1% BSA, was added to each vial. The vials containing cells, unlabeled antibody and radiolabeled antibody were incubated for 1 hour at 4°C. The cells were then harvested by centrifugation (5 min, 1400 g, 4°C) and washed in cold washing buffer (50mM Tris, 150mM NaCl, pH 7.2). The remaining cell-bound radioactivity was measured on a Wizard 3 1480 automatic gamma counter (PerkinElmer Inc., Waltham, MA, USA). Three unharvested vials were counted and served as standards. Non-specific binding was measured by harvesting and counting three vials incubated without cells; the average CPM was subtracted from the data, which was expressed as cell/media CPM. The data was fit to a single-site isotherm (nonlinear regression) model using Prism 7 GraphPad software (GraphPad Inc., San Diego, CA, USA) and an IC_50_ was calculated.

### Xenograft models

All animal experiments were conducted in compliance with institutional guidelines at Memorial Sloan Kettering Cancer Center. Subcutaneous tumors were grafted by injection of 200 μL 1.0-3.0 ×10^6^ cells suspended in a 1:1 v/v mixture of media and Matrigel (Corning Inc., Teterboro, NJ, USA). Male NSG mice (*NOD.Cg-Prkdc SCID IL2rgtm1Wjl/SzJ*), 8-10 weeks old, obtained from the Memorial Sloan Kettering Animal Breeding Facility, were used for K562, THP-1, or BLCL tumors, while LNCaP-AR and BT474 were inoculated in 6-8 weeks old BALB/c (nu/nu) obtained from Charles River Laboratories. Tumors appeared in approximately 2-4 weeks and growth was followed by caliper measurements.

### Biodistribution studies

*In vivo* uptake of ^89^Zr-DFO-MPA1 was evaluated in multiple solid (LNCaP-AR, BT474) and liquid tumor (K562, THP-1, BLCL) models with varying degree of PRAME expression. The radiotracer was administered by tail-vein injection (130–180 μCi, 50 μg of pAb in 100 μL of sterile saline; t = 0 hours) and 4–5 animals were euthanized by CO_2_ asphyxiation at 4, 24, 48 and 72 hours post injection (p.i.). An additional time-point (120 hours p.i.) was added to the study in K562 xenografts (n=5). BLCL mice (n = 4) served as PRAME negative liquid tumor model and were euthanized at 72 hours post injection. To further evaluate *in vivo* targeting specificity a group of THP-1 mice (n = 4) were co-administered with a 20-fold excess of unlabeled MPA1 and euthanized at 24 hours p.i. 14 tissues were collected from all animals, rinsed in water, dried in air, weighted and counted on a Wizard 3 1480 automatic gamma counter (PerkinElmer Inc., Waltham, MA, USA) for accumulation of ^89^Zr-radioactivity (counts per minute, CPM). The count data were background and decay corrected and compared to the total administered dose; expressed as percentage of injected dose per gram (%IA/g, mean ± SD).

### Small-animal positron emission tomography (PET) imaging

Small-animal PET imaging was performed on a microPET Focus 120 Scanner (Concorde Microsystems Inc., Knoxville, TN, USA). Mice were administered ^89^Zr-DFO-MPA1 (120 to 180 μCi; 50 μg of protein in 100μl of sterile saline) through tail vein injection. About 5 min before recording PET images, mice were anesthetized by inhalation of 2% isoflurane (Baxter Healthcare)/oxygen gas mixture and placed on the scanner bed. PET images of K562 xenografts were recorded at 4, 24, 48 and 72 hours post injection, while BLCL xenografts were imaged at 72 hours. List mode data were acquired using a γ-ray energy window of 350 to 750 keV and a coincidence timing window of 6 ns. PET image data were correlated for detector non-uniformity, dead time, random coincidences, and physical decay. No partial volume correction or attenuation correction was applied to the data (in our experience not necessary for quantitative murine imaging). For all images scan time was adjusted to ensure > 20 million recorded events. Sinogram data was reconstructed with a filtered back projection. The images were evaluated by manually delineated volumes of interest (VOIs) applied to the axial views of the data sets (ASIPro, Concorde Microsystems).

### Statistical analysis

Testing for statistical significance was performed using a two-tailed unpaired Student’s *t* test with a 95% confidence interval. In all cases, differences in results were considered to be statistically significant when the computed *P* value was less than 0.05. All analyses were performed using Prism 7 GraphPad software (GraphPad Inc., San Diego, CA, USA).
